# Array Design of 300 GHz Dual-Band Microstrip Antenna Based on Dual-Surfaced Multiple Split-Ring Resonators

**DOI:** 10.3390/s21144912

**Published:** 2021-07-19

**Authors:** Shuhang Bie, Shi Pu

**Affiliations:** Department of Physical Science and Technology, School of Science, Wuhan University of Technology, Wuhan 430070, China; shubiehang@whut.edu.cn

**Keywords:** split-ring resonator, dual-band, microstrip antenna, terahertz

## Abstract

To meet the increasing need of high-data-rate and broadband wireless communication systems, the devices and its circuits R&D under Millimeter, Sub-Millimeter, or even Terahertz (THz) frequency bands are attracting more and more attention from not only academic, but also industrial areas. Most of the former research on the THz waveband (0.1–10 THz) antenna design is mainly focused on realizing high directional gain, such as horn antennas, even though the coverage area is very limited when comparing with the current Wi-Fi system. One solution for the horizontally omnidirectional communication antenna is using the structure of multiple split-ring resonators (MSRRs). Aiming at this point, a novel 300 GHz microstrip antenna array based on the dual-surfaced multiple split-ring resonators (DSMSRRs) is proposed in this paper. By employing the two parallel microstrip transmission lines, different MSRRs are fed and connected on two surfaces of the PCB with a centrally symmetric way about them. The feeding port of the whole antenna is in between the centers of the two microstrip lines. Thus, this kind of structure is a so-called DSMSRR. Based on the different size of the MSRRs, different or multiple working wavebands can be achieved on the whole antenna. Firstly, in this paper, the quasi-static model is used to analyze the factors affecting the resonance frequency of MSRRs. Simulation and measured results demonstrate that the resonant frequency of the proposed array antenna is 300 GHz, which meets the design requirements of the expected frequency point and exhibits good radiation characteristics. Then, a dual-band antenna is designed on the above methods, and it is proved by simulation that the working frequency bands of the proposed dual-band antenna with reflection coefficient below −10 dB are 274.1–295.6 GHz and 306.3–313.4 GHz.

## 1. Introduction

To begin with, we provide a brief background on the terahertz communication. The frequency band of terahertz waves is defined as 0.1–10 THz. Terahertz waves have the characteristics of both microwave and light wave, which have the characteristics of low quantum energy, large bandwidth, and good penetration [1,2,3,4,5]. Therefore, terahertz communication has great development potential and application prospects. Since the electromagnetic wave transmission of 300 GHz has the advantages of lower atmospheric loss, which is widely studied and applied to various communication devices [6,7], 300 GHz is chosen as the simulation frequency of the proposed antenna array. In addition, the 2019 World Radio Communication Conference finally approved a total of 137 GHz of bandwidth resources in the 275–296 GHz, 306–313 GHz, 318–333 GHz, and 356–450 GHz frequency bands for fixed and land mobile service applications [8]. Therefore, 275–296 GHz and 306–313 GHz were selected as the designed frequency bands of the dual-band antenna.

The difficulty of antenna design increases with the improvement of wireless communication requirements, and it is becoming more and more difficult for the current technology to meet the needs of antenna design. Therefore, the unique properties of artificial materials, as applied to antenna, has become an important research direction. As a typical metamaterial structural unit, the split-ring resonator (SRR) can excite magnetic resonance and realize the characteristics of negative permeability. The permeability of a microstrip antenna can be improved effectively by loading SRR, and different functions can be realized according to the properties of the designed metamaterials, such as miniaturized, multi-band, high gain, ultra-wide band, and other advantages. The resonant frequency of its component units is straightforward to adjust and can reach the terahertz frequency band. It has been widely researched and applied in many fields, such as filters [9,10], antennas [11], and energy harvesting [12,13,14,15]. It is shown that the antenna composed of the five split-ring resonators can realize multi-band communication [16].

Designing the antenna array is one of the simple ways to achieve multi-band communication. Array antenna is a kind of special antenna that obtains predetermined radiation characteristics by regular or random arrangement of no less than two antenna elements. Another new high-gain antenna array design method, which improves the directivity of the antenna has been proposed [17]. The reference [18] has proposed an ultra-wideband antenna array design method with good electronic scan performance.

The structure of the paper is as follows. In the first section, the resonance characteristics of MSRRs are analyzed through the quasi-static model. In the second section, a novel microstrip antenna array is proposed for the antenna radiation direction. The 300 GHz frequency is chosen as the resonance frequency of the designed antenna, and the basic structural parameters are determined by the quasi-static model. Then, the electromagnetic simulation software Altair Feko is used to analyze the influence of the antenna’s key size parameters on its resonance frequency, so as to optimize it. Moreover, the feasibility of the design method was verified through simulation and real testing. In the third section, a novel dual-band microstrip antenna is proposed by the above method, and the simulation software is used to optimize the analysis from the two aspects of resonance frequency and bandwidth requirements.

## 2. Analysis of Resonance Characteristics of MSRRs

**Figure 1:**
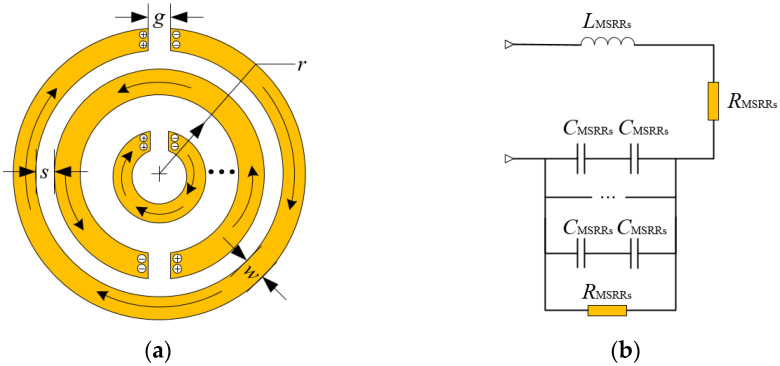
(**a**) Current flow diagram; (**b**) equivalent circuit diagram.

In this section, the characteristics under which MSRRs array is designed are described. When the MSRRs are in a vertically varying magnetic field, the ring current can be generated in the MSRRs, and charges will accumulate at both ends of the space of rings to form the capacitance. Capacitance and inductance (the metal ring can be regarded as the inductance) form a resonant circuit. The quasi-static model is used to analyze it to determine the equivalent circuit when the structural size of the MSRRs is much smaller than the wavelength [19], thereby calculating its resonant frequency. According to the characteristics of the MSRRs, the current flow in MSRRs and its equivalent circuit are shown in Figure 1.

*L*_MSRRs_ is the overall inductance of MSRRs, which is formed by the metal ring inductance connected in series according to the current direction. *C*_MSRRs_ is the overall capacitance of the MSRRs equivalent circuit. *R*_MSRRs_ are equivalent conductor losses, that is, shunt resistance. Its size is related to parameters such as the rings width, *w*, the thickness of the metal patch, and the electrical conductivity of the metal patch. Since two adjacent metal rings can be regarded as one capacitor, the metal rings connected in parallel can be regarded as capacitors connected in parallel. The opening on the ring will still cause the capacitor to be divided into two capacitors in series, which are the *C*_MSRRs_ in the resonant circuit. In addition, due to the small gap between the openings of the metal ring, the capacitance formed by a single metal ring due to the opening can be negligible compared to the capacitance between the rings. Since loop inductances can be connected in series, *L*_MSRRs_ are used to represent the total inductance.

A paper relevant to this research was published, proposing the quasi-static model of MSRRs [20]. According to the quasi-static model theory of MSRRs, the calculation formula of its resonant frequency is derived as shown in Formulas (1)–(4).

(1) $$f=[\sqrt{(C_{\mathrm{MSSRs}}R_{\mathrm{MSRRs}}{}^{2})/L_{\mathrm{MSRRs}}-1}]/(2\pi C_{\mathrm{MSRRs}}R_{\mathrm{MSRRs}})$$

(2) $$C_{\mathrm{MSRRs}}=(n-1)[2r-(2n-1)(w+s)]C_{0}/2$$

(3) $$R_{\mathrm{MSRRs}}=\rho [r-(n-1)(s+w)]/(wt)$$

(4) $$L_{\mathrm{MSRRs}}=4\mu_{0}[r-(n-1)(s+w)][\ln(0.98/\rho )+1.84\rho ]$$

**Figure 2:**
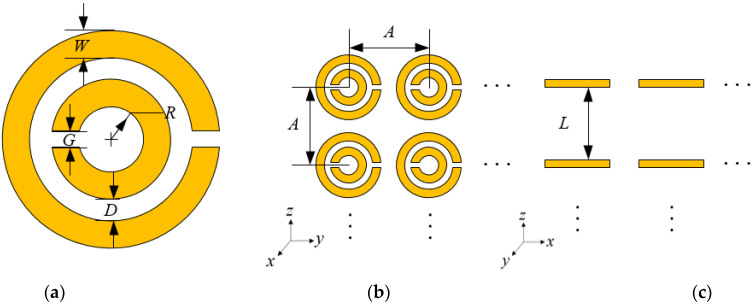
The SRR array: (**a**) the SRR unit; (**b**) the top view; (**c**) the side view.

It can be drawn from the above formula that the number of rings, *n*, the space between rings, *s,* and the width of rings, *w,* are related to the resonance frequency of MSRRs. Moreover, the spacing of the SRR unit also affects the overall radiation performance of the antenna. Therefore, the equivalent permeability formula of the SRR array has certain guiding significance for the subsequent optimization of the distribution form of the antenna array. The reference [21] simply analyzes the mechanism of SRR arrays that can achieve negative permeability. The distribution of the SRR array is shown in Figure 2. The equivalent permeability of the SRR array is finally derived, as shown in Formula (5):

(5) $$\mu_{\mathrm{eff}}=1-\frac{\pi R^{2}}{A^{2}}\cdot \frac{1}{1+j\frac{2L\sigma }{\omega \mu_{0}R}-\frac{2Lc_{0}^{2}}{\omega^{2}\pi R^{3}\ln(2W/D)}}$$

## 3. Design of the 300 GHz Microstrip Array Antenna

**Figure 3:**
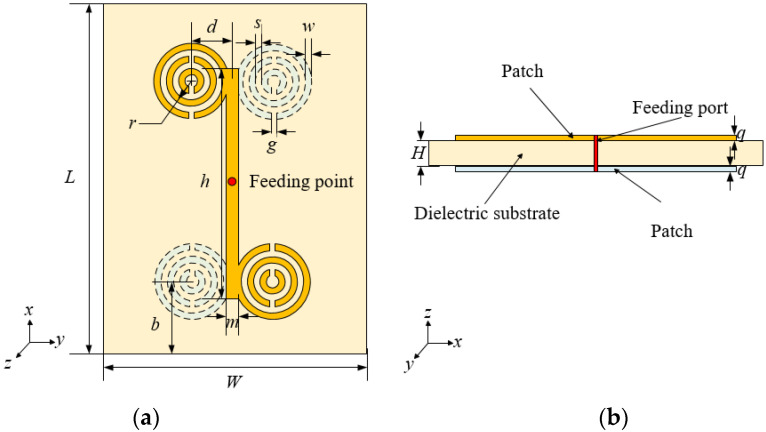
Proposed array antenna with (**a**) the top view; (**b**) the side view.

To increase antenna coverage area, this paper proposes an array antenna. The array antenna is composed of four MSRRs symmetrically printed on the front and back of the dielectric substrate. The epoxy resin material (FR4) of 423 µm × 345 µm × 6.5 µm is used as the dielectric substrate, and its relative dielectric constant is 4.4. The antenna model diagrams are shown in Figure 3. In order to better present the distribution of MSRRs, Figure 3a uses a perspective view to present it. The top metal patch is represented by yellow, the bottom metal patch is represented by white, the dielectric substrate is represented by pink, and the feeding port is represented by the red circle.

The reference [22] has proposed an optimized design of a terahertz microstrip antenna based on DSMSRRs. The basic parameters of the antenna structure can be derived from the above formulas, and then the influence of the main parameters, such as the rings width, *w*, the space between the rings, *s*, and the number of rings, *n,* on the antenna resonance frequency is analyzed by simulation. These results can provide guidance for the optimization of antenna resonant frequency. In addition to the selection of the dielectric plate of the microstrip antenna during the antenna design process, the first consideration is the length of the antenna microstrip lines. In order to satisfy the central resonance frequency of the antenna at 300 GHz, we need to choose a suitable microstrip line length. According to the transmission line theory, using the λ/4 transformability of the transmission line, two transmission lines with different characteristic impedances can be connected. Since the resonant frequency of the designed antenna is 300 GHz, the overall length of the microstrip line is about 500 μm.

### 3.1. The Influence of MSRRs Structure Parameters on Antenna Resonance Frequency

When the radius of outer rings and the distance between the rings are fixed, with the change of the width of rings, *w*, the area of the metal patch will change, which causes the inductance to change [23]. In the analysis of the quasi-static model, the rings width, *w,* also has a direct impact on *C*_MSRR_ and *R*_MSRR_. Therefore, the change of the rings width, *w,* causes some impact in the antenna resonance frequency. We fixed the space between rings, *s,* at 4.4 μm and analyzed the simulation with only the rings width, *w,* changed. Based on the experience, the width of the ring was set to 6.4 μm, 6.8 μm, 7.2 μm, 7.6 μm, 8.1 μm, 8.5 μm, 8.9 μm, and 9.3 μm, and simulations were performed in sequence.

The ratio between the space between the rings, *s,* and the rings’ width, *w,* directly affect the size of the metal area of the MSRRs. Therefore, discussing the effect of the space between rings, *s,* on the antenna resonance frequency is actually to explore the role of this ratio in the antenna performance. We fix the rings width, *w,* at 8.4 μm, and only the size of the space between the rings, *s,* is changed for analysis. Based on experience, the values of space between rings were respectively chosen as 3.07 μm, 3.4 μm, 3.73 μm, 4.06 μm, 4.39 μm, 4.72 μm, 5.05 μm, and 5.38 μm.

For the microstrip array antenna based on DSMSRRs, the number of rings, *n,* will affect the matching of the antenna impedance, and the difference in matching situation have an important influence on the antenna performance. Therefore, we need to match the impedance of the antenna first, and then simulate and analyze the impact of the number of rings, *n,* on the antenna performance. According to the circuit theory, when the characteristic impedance of the antenna and the load impedance are conjugate to each other, there is no power reflection at the feeder terminal, and the antenna achieves maximum power utilization. In this paper, 50 Ω is chosen as the impedance matching standard. After the width and length of the microstrip transmission lines are fixed, matching is performed by adjusting the relative positions of the MSRRs and the microstrip transmission lines.

**Figure 4:**
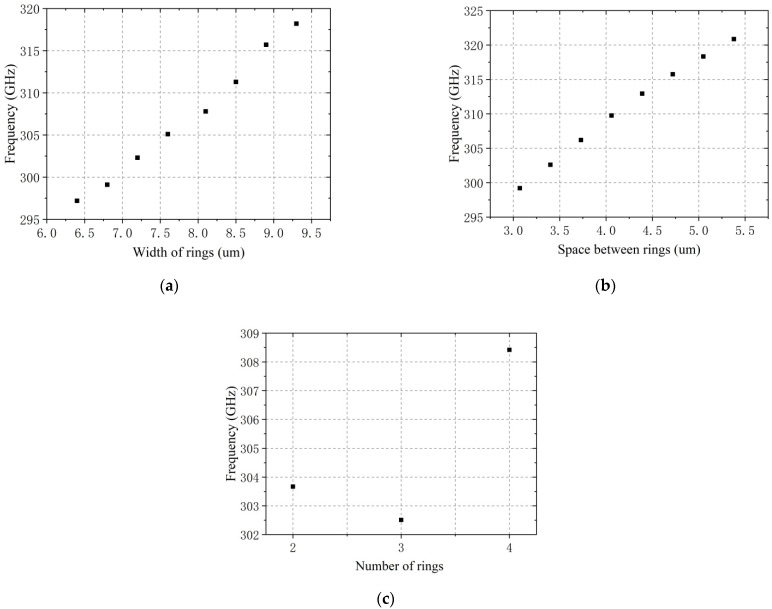
Effect of antenna parameters on resonance frequency: (**a**) width of rings; (**b**) space between rings; (**c**) number of rings.

After the impedance matching is completed, the antenna is simulated and analyzed. Without changing other structural factors, we carried out a qualitative analysis of the width of rings, *w*, the space between rings, *s,* and the number of rings, *n,* through Altair Feko electromagnetic simulation software. The influence of structural parameters on the resonance frequency is shown in Figure 4.

Through simulation analysis, it is concluded that the larger the width of rings, the larger the resonance frequency; when the width of rings is fixed, the larger the space between the rings, and the larger the resonance frequency. Based on this conclusion, empirical values are brought into the simulation calculation, and a scheme with a center frequency point satisfying 300 GHz and good antenna performance is selected. The parameters are shown in Table 1. In addition, this paper uses the MSRRs unit to design the antenna. Although it has the properties of metamaterials, at the same time, because of the particularity of the structure, the precision required by it is higher than that of ordinary THz lens antennas and THz rectangular microstrip antennas.

### 3.2. Simulation Results

To simulate the radiation performance of the antenna in an electromagnetic environment, we used the method of moments (MoM) simulation tool (Altair Feko) to numerically obtain the |*S*_11_| parameters and radiation pattern under the set frequency band.

We first modeled in CADFeko according to the proposed antenna parameters. Among them, it is necessary to establish a line between the top and bottom microstrip transmission lines, then establish the wire feeding port in the middle of the line.

Secondly, taking into consideration that the mesh segment length can affect the time and accuracy of the calculation, it is necessary to set an appropriate segment length. If the mesh segment length is too large, the simulation results will be inaccurate. If the segment length is too small, the results will not converge, and the calculation time will be too long. In general, the segment length is lambda/10. For the subtle parts of the MSSRs structure, the segment length can be smaller in the place where the MSRRs are connected to the microstrip transmission lines; it can be set to lambda/40. In addition, it can also use the high-quality mesh recommended by Altair Feko to divide the model.

**Figure 5:**
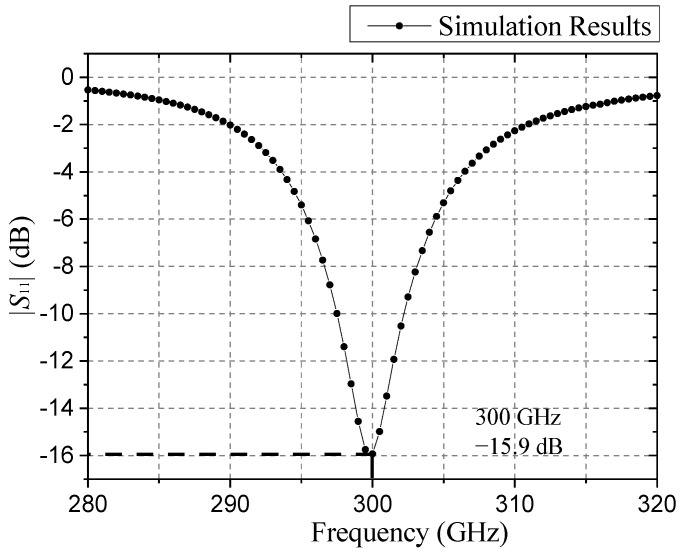
|*S*_11_| of simulation results.

**Figure 6:**
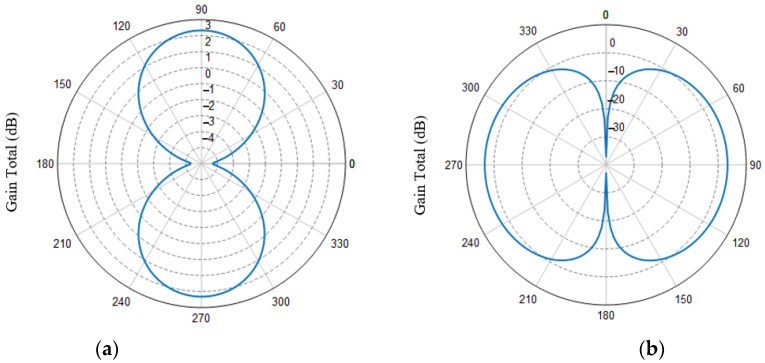
Simulated radiation patterns for (**a**) *E*-plane; (**b**) *H*-plane.

Simulating the optimal structure parameters in Section 3.1, it is concluded that the |*S*_11_|parameter curve is shown in Figure 5. For antennas working in high-frequency bands, the radiation range is an important optimization direction. Figure 6 shows the radiation pattern of the proposed antenna. Compared with the antenna structure in reference [22], the antenna structure proposed in this paper has improved antenna gain and radiation range.

### 3.3. Fabrication of the Proposed Antenna and Measured Results

From the above simulation results, the resonance point of the array antenna is at 300 GHz, and it effectively improves the antenna coverage area. However, because of the limitations of real testing, it is difficult to test the microstrip antenna at 300 GHz. The main difficulties in making the 300 GHz antenna are as follows. For the actual production of the terahertz microstrip antenna designed in this paper, micron-level metal etching needs to be performed on a 423 µm × 345 µm × 6.5 µm dielectric substrate. Therefore, a more precise lithography machine is required for lithography processing, and from waveguide to the microstrip line, it needs a converter which requires special customization. In addition, in the antenna testing, it is also necessary to extend the tool kit to extend the 67 GHz VNA to 300 GHz for measurement. It is difficult to accomplish under current conditions. Therefore, we reduced the resonance frequency of the simulated antenna to an appropriate range by scale effect. We expanded the structural parameters of the array antenna by 300/2.4 times and made tests on this.

The frequency 2.4 GHz belongs to the Industrial Scientific Medical (ISM) band. There are no authorization restrictions for users in this frequency band. Under the condition that the transmitting device meets a certain transmitting power, everyone has the right to use it. In addition, the proposed microstrip antenna has the characteristics of large coverage area and short transmission distance, and is suitable for indoor transmission such as Wi-Fi. The 2.4 GHz band is currently the communication band supported by most home Wi-Fi devices. Considering the practical value of designing the microstrip antenna, we choose the 2.4 GHz band as the test frequency band.

When make the antenna testing, we first made PCB drawings according to the simulation antenna parameters, processed the copper into the MSRRs, and printed them symmetrically on the FR4 substrate. After the production was completed, we selected an SMA connector of 2.4 mm pinhole size, and welded it onto the antenna feeding port, and finally the antenna performance was measured by the AV3656B vector network analyzer of the 41st Institute of China Electronics Technology Group Corporation. Compared with other types of antennas, the microstrip antenna has the advantages of small size, low power dissipation, and simple manufacturing process, and is suitable for mass production.

**Figure 7:**
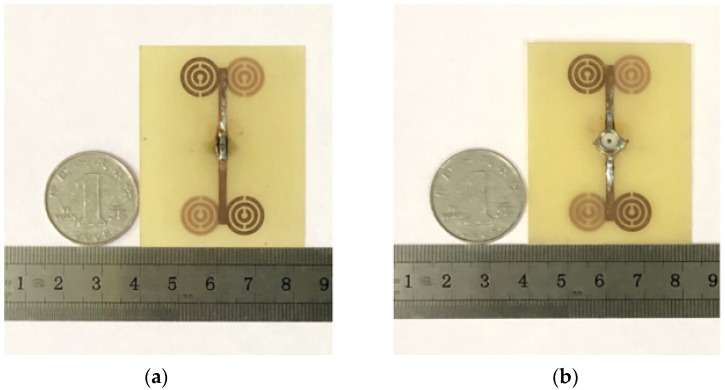
Fabricated antenna model: (**a**) the top view; (**b**) the bottom view.

**Figure 8:**
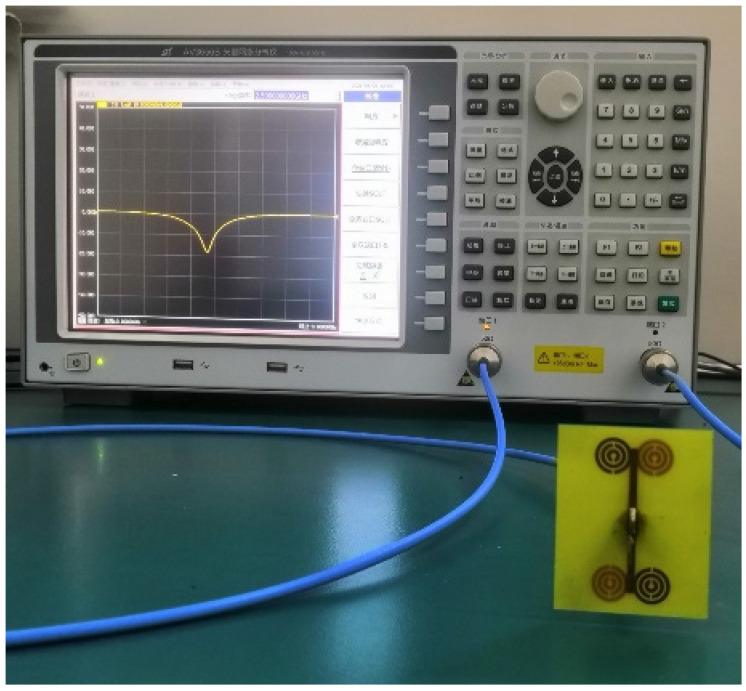
Experimental setup.

**Figure 9:**
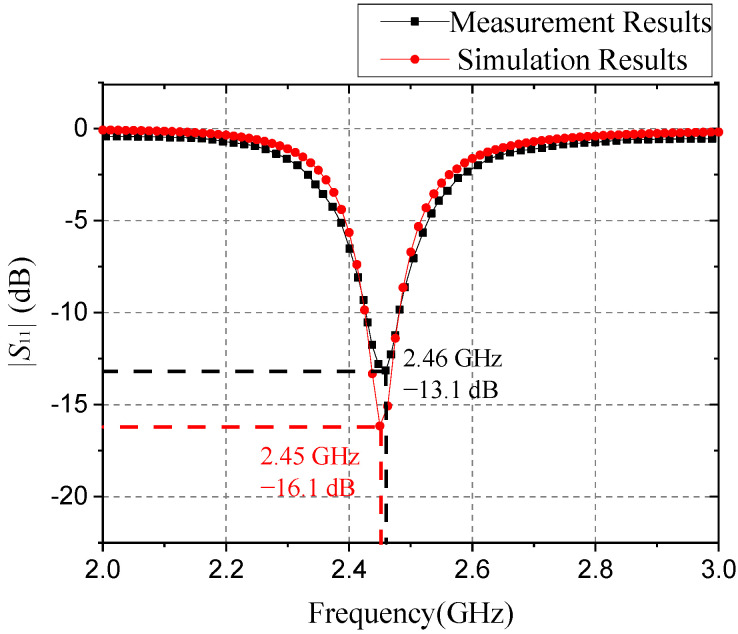
|*S*_11_| of simulation results and measurement results.

The pictures of the antenna model are shown in Figure 7. Figure 7b shows the bottom view of the antenna, which shows the SMA connector welded at the feeding point, and the function of the coin and ruler in the picture is to provide a reference for the size of the fabricated antenna model. Experimental setup is shown in Figure 8, and the simulated and tested |*S*_11_| parameters are shown in Figure 9.

It can be concluded from Figure 9 that the lowest |*S*_11_| parameter in the measurement results reached −13.1 dB, which is a bit worse than the simulation result. In addition, the antenna resonance frequency in the measurement results reached about 2.46 GHz, which is slightly different from the designed 2.45 GHz target. Compared with the simulation process, the SMA connector needs to be welded onto the feeding port of the microstrip transmission lines, which causes a certain deviation in the frequency point of the real testing. In addition, the scale effect and the difference in the antenna production will also cause some errors in the test band. However, when the frequency is expanded to 300 GHz, the error caused by such factors as the SMA connector joint welding will not increase with the scaling. Therefore, we think the error is within the allowable range of the experiment, which verified the rationality and effectiveness of the design of the array antenna structure.

## 4. Design of the 300 GHz Dual-Band Microstrip Antenna

**Figure 10:**
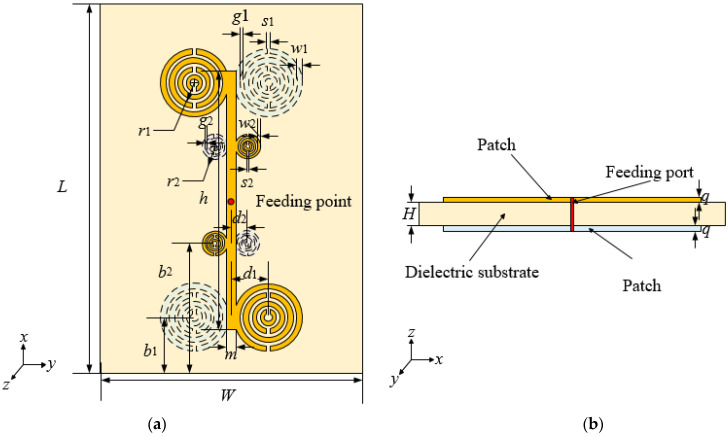
Proposed dual-band antenna with (**a**) the top view; (**b**) the side view.

Based on the above design method, this paper proposes a novel dual-band antenna array, which can meet the needs of communication in different frequency bands. Based on the structure of the designed antenna array and the quasi-static model analysis method, we designed two array antennas of four split-ring resonators and three split-ring resonators that can basically meet the requirements of 275–296 GHz and 306–313 GHz bands. The antenna is composed of three split-ring resonators and four split-ring resonators symmetrically printed on the front and back sides of the dielectric substrate, thereby achieving the purpose of dual-band communication in the terahertz waveband. Diagrams of the dual-band antenna model are shown in Figure 10.

### 4.1. Optimization of Dual-Band Microstrip Antenna Structure Parameters

The addition of the three split-ring resonators structure will disturb the current distribution on the surface of the patch, thereby affecting the performance of the antenna. Therefore, the dual-band antenna structure still needs to be properly optimized to increase its communication bandwidth. There are many methods to increase the bandwidth of microstrip antennas, such as slotting the antenna structure to change the current path [24,25,26], loading short-circuit probes [27], attaching parasitic patches [28], changing the thickness and dielectric of the dielectric substrate electric constant [29], and so on.

Since the dielectric substrate of the microstrip antenna was selected as the epoxy resin material, we adopted the method of increasing the thickness of the dielectric substrate to improve the bandwidth of the antenna. The quality of the impedance matching result also affects the antenna performance. Therefore, we made appropriate adjustments to the antenna’s microstrip transmission lines width and the relative position of the MSRRs and the microstrip transmission lines to improve the antenna matching, and thus expand the antenna bandwidth. After comprehensively considering some key parameters of the antenna, we chose a solution that satisfies and matches the best bandwidth. Finally, the structural parameters of the dielectric substrate were determined to be 667 µm × 544 µm × 20 µm. The key size parameters of the antenna are shown in Table 2.

### 4.2. Simulation Results

**Figure 11:**
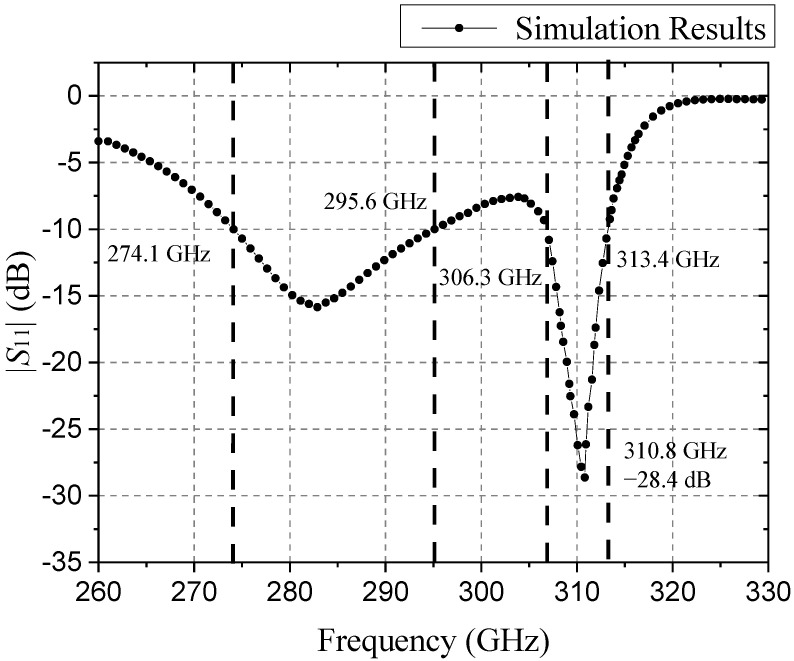
|*S*_11_| of simulation results.

**Figure 12:**
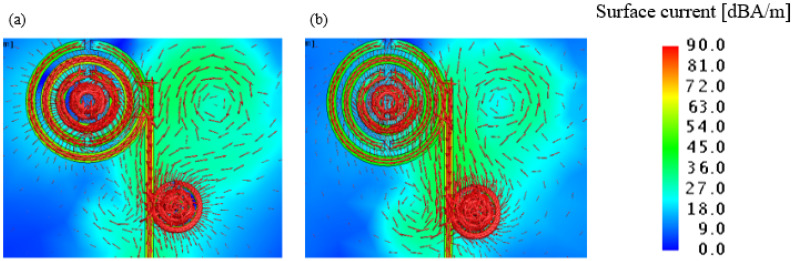
Simulated surface current distribution: (**a**) 282 GHz; (**b**) 310 GHz.

Based on the above optimized structural parameter simulation, the dual-band antenna |*S*_11_| parameter curve is shown in Figure 11. The current distribution of the MSRRs is shown in Figure 12.

From the above simulation results, the effective bandwidth of the antenna is 274.1–295.6 GHz and 306.3–313.4 GHz, and the lowest |*S*_11_| parameter reached −28.4 dB, which basically meets the design requirements. When the dual-band antenna is working, the current will first flow through the three split-ring resonators structure, and then through the four split-ring resonators structure, so this will cause loss of |*S*_11_| performance parameters in the first band (274.1–295.6 GHz).

Since the dual-band antenna adopts the abovementioned array antenna structure, the radiation direction of the entire antenna is effectively improved. In addition, when the resonance point is around 282 GHz, the current distribution in the four split-ring resonators is relatively large, and when the resonance point is around 310 GHz, the current distribution in the four split-ring resonators is relatively small, while the current distribution in the three split-ring resonators is relatively large, which further proves the rationality of the antenna design.

## 5. Conclusions

In this paper, a 300 GHz microstrip antenna array is proposed based on the DSMSRRs. Directed by quasi-static analysis mode, the simulation software was used to simulate and analyze the influence of the antenna resonant frequency from the three aspects of the width of rings, the space between rings, and the number of rings. In addition, 300 GHz was set as the resonant frequency for simulation and real testing, which verifies the validity of the design method and demonstrates good antenna radiation performance. Then, based on the above design method, we optimized two kinds of MSRRs and combined them into a new dual-band microstrip antenna. After considering the influence of dielectric plate thickness and impedance matching on the antenna bandwidth, we optimized and simulated the dual-band antenna. The simulation results show that the microstrip antenna can meet the bandwidth requirements of 274.1–295.6 GHz and 306.3–313.4 GHz. This paper provides a new method for realizing multi-band communication by loading multiple MSRRs with different resonant frequencies at both ends of the feeding lines.

## Figures and Tables

**Table 1:** Parameters of the optimized array antenna.

Name	Value (μm)	Name	Value (μm)
*r*	41.20	*h*	326.00
*s*	4.72	*d*	46.00
*g*	4.70	*b*	62.00
*m*	20.00	*w*	8.50

**Table 2:** Parameters of the optimized array dual-band antenna.

Name	Value (μm)	Name	Value (μm)
*r*_1_	72.50	*r*_2_	30.00
*w*_1_	11.00	*w*_2_	6.00
*s*_1_	6.30	*s*_2_	3.40
*g*_1_	6.30	*g*_2_	3.40
*d*_1_	72.50	*d*_2_	30.00
*b*_1_	102.80	*b*_2_	229.00
*M*	10.00	*h*	514.00

## Data Availability

Not applicable.

## Nomenclature

DSMSRR	dual-surfaced multiple split-ring resonators
MSRRS	multiple split-ring resonators
SRR	split-ring resonators
